# Thymosin α1 combined with immune checkpoint inhibitors: synergistic remodeling of the tumor immune microenvironment to enhance clinical efficacy and safety

**DOI:** 10.3389/fimmu.2026.1762151

**Published:** 2026-05-29

**Authors:** Haiyv Guo, Ruichao Li

**Affiliations:** Department of Geriatrics, Tongji Hospital, Tongji Medical College, Huazhong University of Science and Technology, Wuhan, China

**Keywords:** enhanced efficacy, immune checkpoint inhibitors, immune-related adverse events, safety, synergistic effect, thymosin α1

## Abstract

Although immune checkpoint inhibitors (ICIs) have revolutionized the therapeutic landscape of solid tumors, their clinical utility remains constrained by several challenges, including tumor heterogeneity, immunosuppressive tumor microenvironments, and immune-related adverse events (irAEs), which collectively limit overall response rates. Thymosin α1 (Tα1), a pleiotropic immunomodulator, not only enhances immune competence and regulates excessive immune activation but also exerts direct antitumor effects. Given these immunoregulatory and antitumor properties, a growing body of preclinical and clinical studies has investigated the combination of Tα1 with ICIs, demonstrating its potential to augment the efficacy of ICIs and mitigate their limitations. In this review, we summarize and discuss the biological characteristics of Tα1 and current evidence regarding the synergistic effects of Tα1 combined with ICIs. Preliminary findings suggest that this combination exhibits promising clinical efficacy with manageable safety profiles in cancer therapy. Nevertheless, large-scale and long-term clinical studies are warranted to further validate its sustained clinical benefits.

## 1 Background

### 1.1 Limitations of immune checkpoint inhibitors in solid tumor therapy: immunosuppressive microenvironment and mechanisms of resistance

The clinical application of ICIs has led to a paradigm shift in cancer therapy, marking a revolutionary advance following chemotherapy and targeted therapy. Despite their remarkable and durable antitumor effects in a subset of patients, the overall response rate to ICIs remains modest. Moreover, severe immune-related adverse events (irAEs) may compromise treatment tolerance and long-term outcomes.

Multiple factors contribute to the limited efficacy of ICIs in solid tumors.

1. Tumor heterogeneity: Tumor cells display marked genetic and phenotypic diversity, and immune microenvironments vary substantially across tumor types. Consequently, lymphocytes can exert either tumor-promoting or tumor-suppressive effects depending on the tumor context.
2. Immunosuppressive microenvironment: The tumor microenvironment (TME) is often enriched with immunosuppressive cell populations such as myeloid-derived suppressor cells (MDSCs) and M2-polarized macrophages, which secrete inhibitory cytokines including IL-10 and TGF-β, thereby dampening T-cell activity (1, 2).
3. Insufficient tumor-infiltrating lymphocytes (TILs): Deficiency or functional impairment of TILs gives rise to “cold tumors.” Approximately 60% of solid tumors lack adequate CD8^+^ T-cell infiltration, rendering them unresponsive to ICIs (3, 4).
4. Acquired resistance during treatment: This process involves upregulation of multiple T-cell exhaustion markers (e.g., TIM-3, LAG-3) and compensatory expansion of immunosuppressive cell subsets such as regulatory T cells (Tregs) and M2-type tumor-associated macrophages (5, 6). Recent studies further implicate aberrant activation of the IL-1β signaling pathway as a key molecular driver of immunosuppressive TME formation, promoting MDSC recruitment and M2 polarization, and ultimately leading to a T-cell–excluded phenotype (1, 7).
5. Epigenetic dysregulation: Epigenetic alterations can impair T-cell function. For instance, METTL3-mediated m^6^A RNA methylation modulates immune checkpoint molecule expression, thereby accelerating T-cell exhaustion (5).
6. Metabolic reprogramming within the TME: Metabolic competition between tumor cells and effector lymphocytes contributes to immune suppression. Tumor cells depend on the Warburg effect and glutamine metabolism, depleting essential nutrients required for T-cell function. Under hypoxic conditions, HIF-1α-driven glycolysis promotes lactate accumulation and acidification, creating a self-reinforcing suppressive environment (8–10).

### 1.2 Multifaceted immunomodulatory effects of thymosin α1: remodeling TME

**Figure 1:**
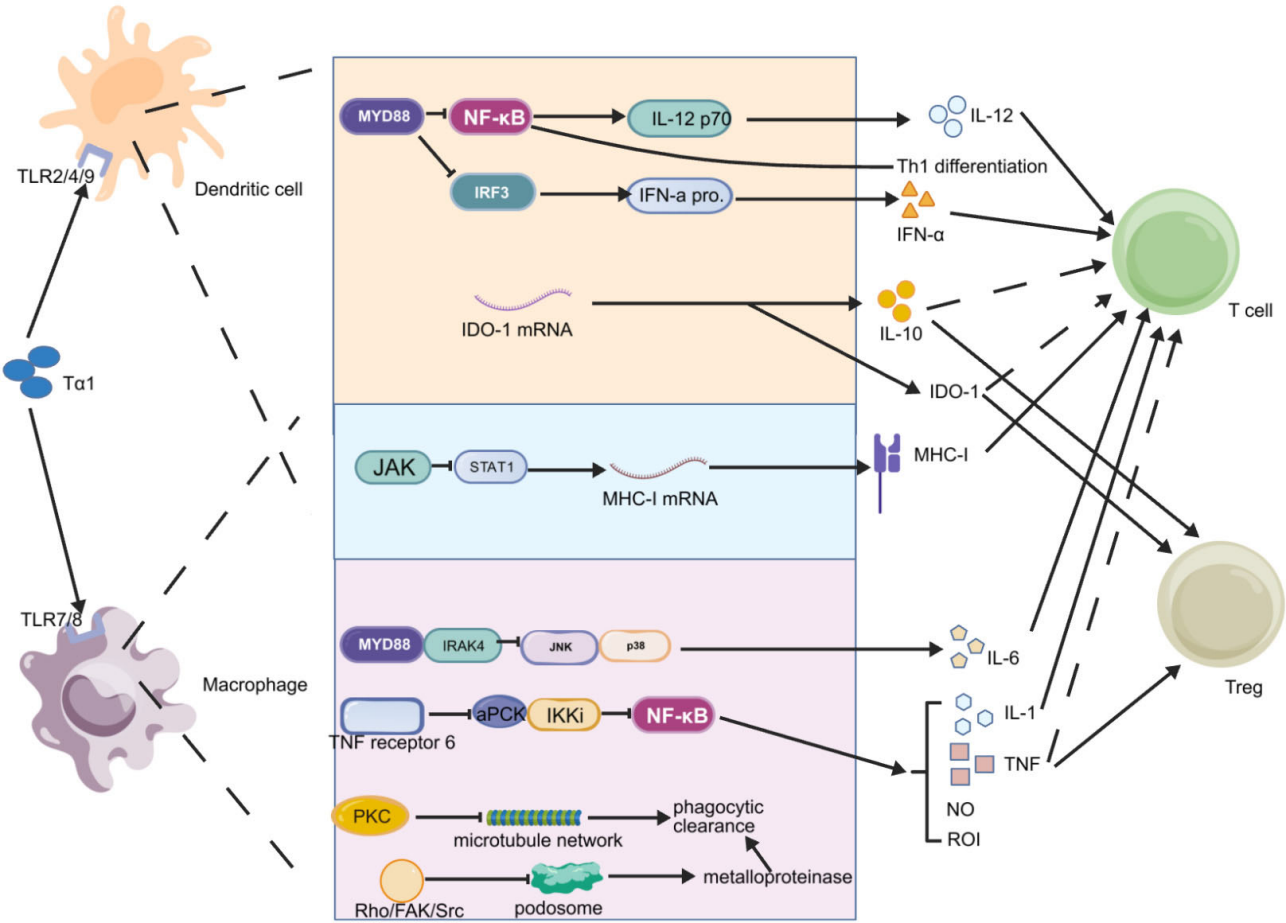
Regulation of the innate immune system by Tα1. The dashed arrows represent inhibition, while the solid arrows represent promotion. Tα1, Thymalfasin (Thymosin Alpha-1); TLR, Toll-like Receptor; PKC, Protein Kinase C; Rho, Rho Family GTPases; FAK, Focal Adhesion Kinase; Src, Family Kinases; IDO-1, Indoleamine 2, 3-Dioxygenase 1; IL-12, Interleukin-12; IL-1, Interleukin-1; IL-10, Interleukin-10; Th1, T Helper Type 1; IFN-α, Interferon-Alpha; TNF, Tumor Necrosis Factor; NO, Nitric Oxide; ROI, Reactive Oxygen Intermediate; Treg, Regulatory T Cell.

Tα1 is a synthetic 28-amino acid peptide with pleiotropic and multitargeted immunomodulatory activities (**Figure 1**). It enhances lymphoid function, suppresses excessive inflammatory responses, and restores immune homeostasis. Approved by the U.S. Food and Drug Administration (FDA), Tα1 is recommended in clinical guidelines for the management of malignant melanoma, hepatocellular carcinoma, viral hepatitis, and various immunodeficiency disorders (11–14).

During the COVID-19 pandemic, Tα1 demonstrated notable clinical efficacy by restoring immune function and preventing lymphocyte depletion through suppression of cytokine storms, promotion of lymphocyte proliferation, and enhancement of T-cell activity (14, 15). Previous studies also indicate that Tα1 possesses direct antitumor activity by inhibiting tumor cell proliferation and inducing apoptosis.

Recent investigations have explored Tα1 as an adjuvant to chemotherapy, revealing its ability to potentiate conventional anticancer regimens (16). In stage IIIA non–small cell lung cancer (NSCLC), Tα1 co-administration significantly improved survival outcomes, with a 14.3% increase in the 5-year survival rate (hazard ratio = 0.72, p = 0.032) (17, 18). Consequently, Tα1 has been widely adopted as an adjunctive agent in clinical chemotherapy protocols. Moreover, in combination with radiotherapy, Tα1 was shown to markedly reduce radiation-induced inflammatory responses (19). Several studies have further investigated its postoperative adjuvant use in cancer patients, demonstrating reduced postoperative complications and improved long-term survival (20).

### 1.3 Promising prospects for combination therapy

As research advances in the understanding of the immunosuppressive tumor microenvironment and the mechanistic limitations of ICIs, there is an urgent need for therapeutic strategies that can enhance the overall response rate to ICIs while mitigating their associated adverse effects. Given the compelling preclinical and clinical evidence supporting the dual immunoregulatory and antitumor functions of Tα1, combining Tα1 with ICIs represents a rational and promising approach. This strategy is anticipated to potentiate antitumor immune responses, alleviate immune-related toxicities, and ultimately improve patient outcomes.

## 2 Mechanism by which thymalfasin counteracts irAEs

ICIs primarily target CTLA-4, PD-1, and PD-L1 molecules. CTLA-4 and PD-1 are mainly expressed on the surface of T lymphocytes (21, 22), whereas PD-L1 is predominantly expressed on tumor cells (23, 24). In addition, these molecules are also expressed on normal tissue cells, where they play a critical role in maintaining self-immune tolerance. Consequently, blockade of immune checkpoints disrupts normal immune tolerance mechanisms, leading to the development of adverse events. Among these, CTLA-4 inhibitors are associated with a higher incidence of adverse events (25, 26), largely because PD-1/PD-L1 inhibitors exhibit a more restricted effect within TME (21).

Blockade of CTLA-4, PD-1, and PD-L1 enhances the activation and proliferation of CD4^+^ and CD8^+^ T cells, accompanied by increased release of proinflammatory cytokines such as TNF, IFN-γ, and IL-2 (27–30). In addition, depletion and impaired generation of Tregs (31–34) disrupt immune homeostasis in normal tissues, resulting in tissue damage. Studies have shown that CTLA-4 inhibitors may increase the number of Th17 cells in patients with cancer, thereby contributing to the development of colitis (35, 36). Furthermore, immune checkpoint blockade can induce the production of autoantibodies and cross-reactive antigens (37–41), which may cause damage to normal tissues.

Tα1 has been shown to upregulate indoleamine 2, 3-dioxygenase 1 (IDO-1) mRNA expression outside the tumor, thereby enhancing tryptophan metabolism and suppressing T lymphocyte metabolism, ultimately inhibiting T cell function. In addition, Tα1 promotes the activation and proliferation of CD4^+^CD25^+^Foxp3^+^ Tregs by modulating signaling pathways in peripheral blood mononuclear cells (42). Collectively, these mechanisms suggest that Tα1 may reduce the incidence of adverse events resulting from excessive immune cell activation induced by ICIs in normal tissues.

## 3 Molecular mechanisms by which Tα1 remodels the tumor immune microenvironment

**Figure 2:**
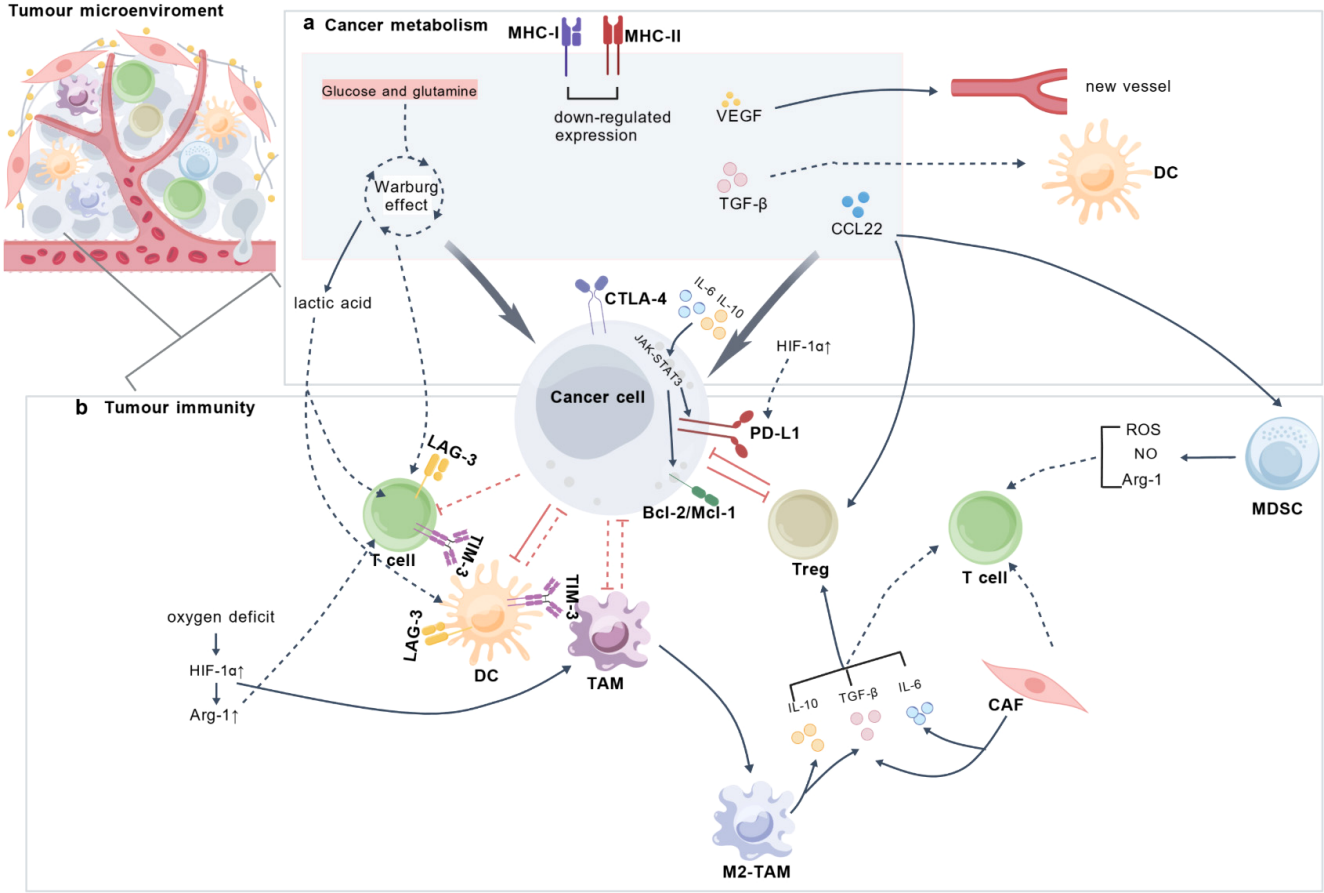
Mechanisms of immunosuppression and the tumor microenvironment. The dashed arrows represent inhibition, while the solid arrows represent promotion. DC, Dendritic Cell; NK cell, Natural Killer cell; MDSC, myeloid-derived suppressor cell; TAM, Tumor-Associated Macrophage; M2-TAM, M2-polarized Tumor-Associated Macrophage; Treg, Regulatory T Cell; VEGF, Vascular Endothelial Growth Factor; TGF-β, Transforming Growth Factor-β; MHC-I, Major Histocompatibility Complex Class I; MHC-II, Major Histocompatibility Complex Class II; CCL22, C-C Motif Chemokine Ligand 22 C-C; IL-6, Interleukin-6; IL-10, Interleukin-10; ROS, Reactive Oxygen Species; NO, Nitric Oxide; Arg-1, Arginase 1; CAF, Cancer-associated fibroblast; LAG-3, lymphocyte activation gene3; TIM-3, T-cell immunoglobulin domain and mucin domain-3 T; HIF-1α, Hypoxia-Inducible Factor-1 α; PD-1, Programmed Cell Death Protein 1; PD-L1, Programmed Death-Ligand 1; CTLA-4, Cytotoxic T-Lymphocyte Associated Protein 4; Bcl-2, B-Cell Lymphoma 2.

**Figure 3:**
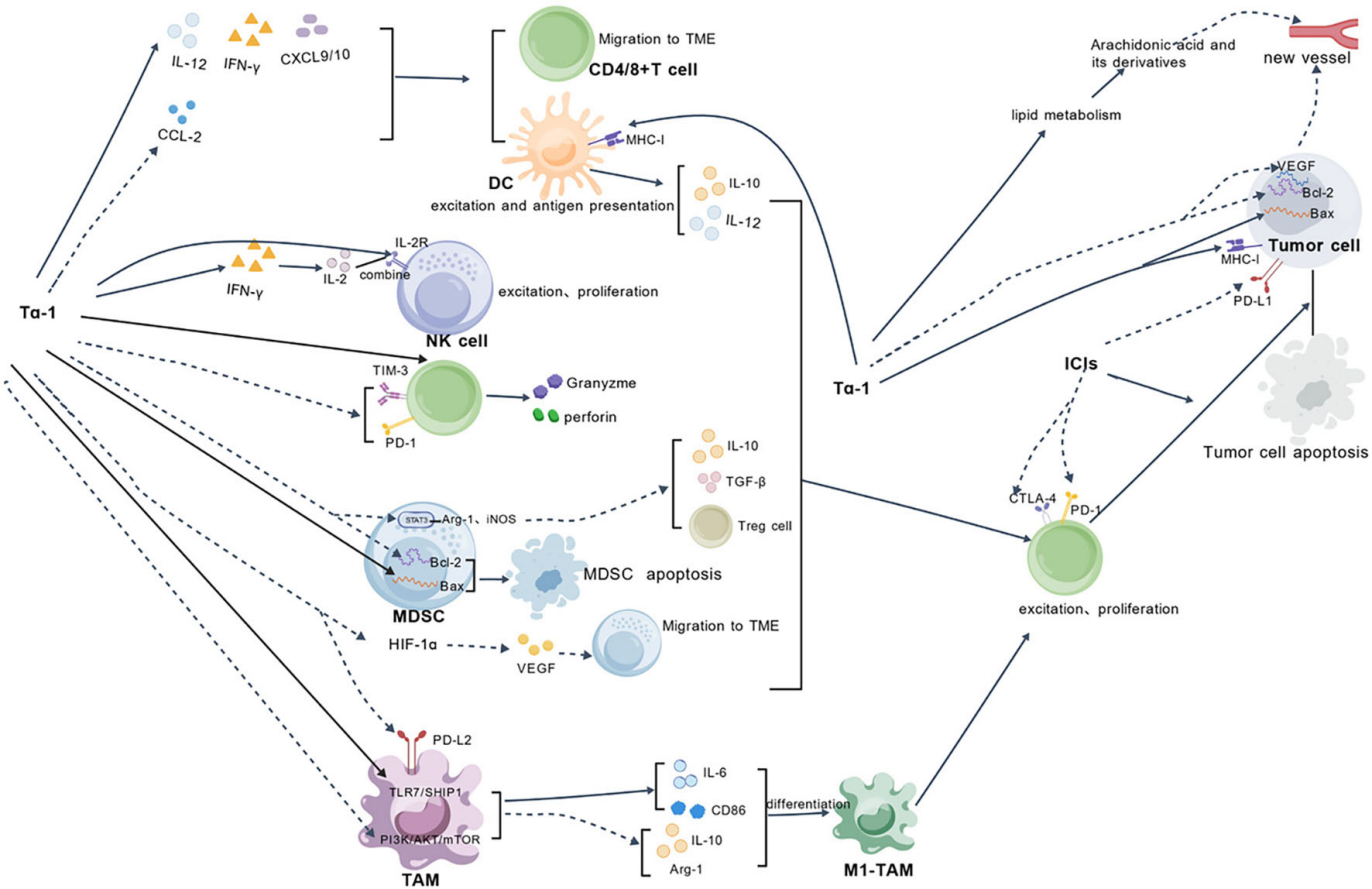
Regulatory effects of Tα1 in Tumor immune microenvironment in synergy with ICIs-mediated antitumor activity. The dashed arrows represent inhibition, while the solid arrows represent promotion. Tα1, Thymalfasin (Thymosin Alpha-1); IL-12, Interleukin-12; IFN-γ, Interferon-gamma; CXCL9/10, C-X-C motif chemokine ligand 9/10; CCL22, C-C Motif Chemokine Ligand 22 C-C; TME, Tumor Microenvironment; DC, Dendritic Cell; MHC-I, Major Histocompatibility Complex Class I; IL-10, Interleukin-10; IL-2, Interleukin-2; IL-2R, Interleukin-2 receptor; NK cell, Natural Killer cell; TIM-3, T-cell immunoglobulin domain and mucin domain-3 T; PD-1, Programmed Cell Death Protein 1; Arg 1, Arginase 1; iNOS, Inducible Nitric Oxide Synthase; Bcl-2, B-Cell Lymphoma 2; Bax, BCL2-Associated X Protein; MDSC, myeloid-derived suppressor cell; HIF-1α, Hypoxia-Inducible Factor-1 Alpha; VEGF, Vascular Endothelial Growth Factor; IL-2, Interleukin-6; CD86, Cluster of Differentiation 86; TGF-β, Transforming Growth Factor-β; Treg, Regulatory T Cell; TAM, Tumor-Associated Macrophage; M1-TAM, M1-polarized Tumor-Associated Macrophage; PD-L1, Programmed Death-Ligand 1; PD-L2, Programmed Death-Ligand 2; ICIs, Immune Checkpoint Inhibitors; CTLA-4, Cytotoxic T-Lymphocyte Associated Protein 4.

Tα1 exerts multifaceted immunoregulatory effects that target multiple molecular pathways to reverse the immunosuppressive TME while simultaneously exhibiting direct cytotoxic activity against tumor cells (**Figures 2**, **3**). The mechanistic insights are summarized as follows:

### 3.1 Targeting and depleting immunosuppressive cell populations

#### 3.1.1 Suppression of MDSC function and reduction of Treg infiltration

In NSCLC and other tumor models, MDSCs suppress T-cells activity and promote Tregs accumulation through the secretion of inhibitory cytokines (e.g., IL-10, TGF-β) and depletion of arginine metabolism. Tα1 effectively inhibits the STAT3 signaling cascade, thereby downregulating the expression of arginase-1 (Arg-1) and inducible nitric oxide synthase (iNOS). This blockade markedly attenuates the immunosuppressive activity of MDSCs and indirectly limits Treg recruitment within tumor tissue, leading to a 3.2-fold increase in the intratumoral CD8^+^/Treg ratio and reversal of the immunosuppressive TME (43, 44).

In addition, Tα1 has been shown to downregulate the expression of the anti-apoptotic gene Bcl-2 and upregulate the pro-apoptotic gene Bax in MDSCs, thereby promoting MDSC apoptosis (43). Furthermore, Tα1 inhibits the expression of hypoxia-inducible factor-1α (HIF-1α) under tumor hypoxic conditions, reducing the production of the MDSC chemokine vascular endothelial growth factor (VEGF), which in turn suppresses the specific migration of MDSCs into TME (43).

#### 3.1.2 Reprogramming macrophage polarization

Tα1 recognizes and binds to phosphatidylserine (PS) on the surface of apoptotic cells and is subsequently internalized by macrophages. This triggers activation of the TLR7/SHIP1 signaling axis and suppression of the PI3K/AKT/mTOR pathway (45), resulting in upregulated the expression of IL-6 and CD86 while downregulating the expression of IL-10 and Arg-1, thereby blocking the polarization of M2-type tumor-associated macrophages (TAMs). The M1/M2 ratio increases significantly from 0.3 to 1.8 (p < 0.01) (45, 46).

### 3.2 Enhancement of antitumor immune responses

#### 3.2.1 Promotion of dendritic cell activation and function

Tα1 can upregulate the expression of Th1-type cytokines such as IL-12 and IFN-γ within the tumor microenvironment (TME), promoting CXCL9/CXCL10 secretion while suppressing CCL22, thereby enhancing dendritic cell (DC) antigen-presenting capacity and facilitating T-cell trafficking to tumor sites (47). Studies in fungal infection and inflammatory contexts have shown that Tα1 activates DCs, inducing IL-12 release, while also activating plasmacytoid DCs and promoting IL-10 expression, which further stimulates CD8^+^ T-cell activation (48, 49). Mature DCs effectively cross-present tumor antigens, thereby activating CD8^+^ T cells and enhancing their cytotoxic function.

#### 3.2.2 Upregulation of MHC molecule expression and enhancement of NK cell activity

Through activation of the JAK/STAT and interferon regulatory factor (IRF) signaling pathways, Tα1 significantly increases tumor cell surface MHC-I molecule expression, thereby improving CD8^+^ T-cell recognition and cytotoxic clearance of tumor cells (46, 50). In multiple tumor models, immunosuppressive contexts, and *in vitro* studies, Tα1 has been shown to enhance NK cell activity and numbers, thereby inhibiting tumor cell proliferation and promoting apoptosis (51–54). Garaci E et al. demonstrated in both immunosuppressive and tumor models that Tα1 upregulates IL-2 and its NK cell surface receptor expression, and through enhanced IFN-γ expression, facilitates IL-2/receptor interactions, thereby initiating NK cell activation (55).

#### 3.2.3 Direct promotion of T lymphocyte function

Yf L et al. reported in a hepatitis B context that Tα1 upregulates T-cell receptor (TCR) expression on T lymphocytes, leading to activation of Lck/ZAP-70 signaling, which promotes T-cell proliferation and functional activity. Additionally, Tα1 activates the MAPK signaling pathway within T lymphocytes, further enhancing their proliferation and activation (56). Non-tumor studies have also shown that Tα1 can downregulate senescence-related markers on CD8^+^ T cells (PD-1, TIM3), thereby extending their lifespan (57). In lung cancer mouse models, Tα1 upregulates CD8^+^ T-cell cytotoxic markers, perforin and granzyme, promoting T-cell-mediated antitumor immunity (48). However, the precise mechanisms by which Tα1 affects specific T-cell subsets within the TME remain incompletely defined.

### 3.3 Direct effects on tumor cells

#### 3.3.1 Promotion of tumor cell apoptosis

In addition to its indirect effects within TME—including promoting DC activation, modulating macrophage polarization, inhibiting MDSCs, enhancing NK cell function, and activating CD8^+^ T cells through cytokine regulation to promote proliferation and enhance tumor cell cytotoxicity—Tα1 also exerts direct effects on tumor cells to induce apoptosis. Specifically, Tα1 acts directly on tumor cells, leading to a decrease in the proportion of cells in the S phase of the cell cycle and thereby inhibiting tumor cell proliferation (58). In breast cancer studies, Tα1 has been shown to upregulate PTEN protein expression and inhibit the PI3K/Akt/mTOR signaling pathway, triggering the mitochondrial apoptotic pathway, as evidenced by decreased mitochondrial membrane potential, increased Bax/Bcl-2 ratio, and activation of caspase-3/9 and PARP, ultimately promoting tumor cell apoptosis (59). In hematologic malignancies, Tα1 directly suppresses Bcl-2 expression and upregulates Fas/Apo-1 expression, exerting a direct cytotoxic effect on tumor cells; however, this mechanism was found to be ineffective in other tumor types (60).

#### 3.3.2 Inhibition of angiogenesis

Experimental studies demonstrate that Tα1 binds to specific receptors on tumor cell membranes, activating the phospholipase C(PLC) signaling cascade. This leads to hydrolysis of phosphatidylinositol-4, 5-bisphosphate (PIP_2_) into inositol triphosphate (IP_3_) and diacylglycerol (DAG), which subsequently activate protein kinase C (PKC). PKC-mediated metabolism generates arachidonic acid and its derivatives (e.g., prostaglandins and thromboxanes). These lipid mediators can effectively inhibit tumor angiogenesis, thereby further promoting tumor cell apoptosis (61).

### 3.4 Regulation of immune metabolism

#### 3.4.1 Reversal of tumor hypoxia

Tα1 inhibits hypoxia-inducible factor 1α (HIF-1α) transcriptional activity, thereby reducing the expression of downstream targets such as VEGF and PD-L1. This contributes to vascular normalization, alleviation of hypoxia, and suppression of HIF-1α–mediated MDSC recruitment and T-cell inhibition (62, 63).

#### 3.4.2 Modulation of tryptophan metabolism

Animal studies have shown that Tα1 enhances peripheral indoleamine 2, 3-dioxygenase-1 (IDO-1) activity, promoting the conversion of tryptophan to kynurenine and increasing the kynurenine/tryptophan ratio. Kynurenine, in turn, facilitates Treg differentiation and MDSC expansion, suppresses the mTOR signaling pathway, and thereby exerts negative feedback on T-cell activation. This modulation prevents excessive immune activation and mitigates immune-mediated tissue injury (8, 47, 64).

## 4 Mechanistic rationale and feasibility of the combination strategy

The synergistic antitumor effects observed with the combination of Tα1 and immune ICIs are supported by solid molecular and cellular foundations. This synergy manifests across three mechanistic dimensions—spatial, temporal, and metabolic coordination—each contributing to enhanced therapeutic efficacy.

### 4.1 Spatial synergy

Tα1 alleviates the TME by reducing the density of MDSCs and the proportion of M2-type TAMs. These changes facilitate cytotoxic T lymphocyte (CTL) infiltration and reactivation within the tumor bed, thereby converting an immune-excluded milieu into an inflamed, immune-permissive state (8, 10).

### 4.2 Temporal synergy

Preconditioning with Tα1 upregulates major histocompatibility complex class I (MHC-I) expression on tumor cells by approximately 2.1-fold, which enhances subsequent ICI-mediated immune recognition and tumor-specific cytotoxicity (65, 66). This priming effect increases both the sensitivity and specificity of ICI treatment, leading to more robust antitumor responses.

### 4.3 Metabolic modulation synergy

Tα1 regulates immune metabolism through dual mechanisms. First, by suppressing Arg-1 activity, Tα1 mitigates arginine depletion, thereby restoring T-cell metabolic fitness and effector function (5, 45). Second, by upregulating indoleamine IDO activity in non-tumor tissues, Tα1 modulates tryptophan metabolism to prevent excessive T-cell activation and limit irAEs.

Collectively, these multimodal mechanisms provide a strong biological rationale for the combined use of Tα1 and ICIs. This combination holds promise for reprogramming “cold” tumors into “hot” immunologically active phenotypes, thereby enhancing response rates to immunotherapy and expanding its clinical benefit (62, 67, 68).

## 5 Synergistic effects and clinical survival benefits of Tα1 combined with ICIs

### 5.1 Preclinical evidence: Tα1 enhances the antitumor efficacy and mitigates the toxicity of ICIs

Giorgia Renga et al. (47) demonstrated in murine models that Tα1 can restore innate immune homeostasis disrupted by dextran sulfate sodium (DSS), which induces colonic injury via innate immune activation. Co-administration of Tα1 with the CTLA-4 inhibitor further confirmed its protective role against immune-mediated intestinal damage. Compared with DSS-only mice, the combination group showed markedly reduced immune-related intestinal toxicity, with histopathology revealing restored mucosal barrier integrity and improved intestinal length.

Building upon these findings, the investigators confirmed that Tα1 combined with ICIs provides consistent protection against intestinal toxicity without compromising the antitumor activity of the ICIs. In a melanoma-bearing mouse model, the combination of CTLA-4 blockade with Tα1 produced a pronounced synergistic effect, characterized by suppressed tumor proliferation and enhanced tumor necrosis. Subsequent studies extended these findings to Tα1 combined with PD-1 blockade in melanoma and lung adenocarcinoma models, both showing significant antitumor potentiation.

Mechanistically, Tα1 was found to upregulate indoleamine IDO 1 mRNA expression in the peritumoral microenvironment, increasing Foxp3^+^ Treg levels while reducing proinflammatory cytokine secretion, thereby promoting immune tolerance. In contrast, Tα1 did not increase IDO1 expression within tumor tissue itself; instead, it enhanced CD4^+^ and CD8^+^ T cell infiltration while reducing intratumoral Treg frequency—collectively shifting the immune milieu toward a cytotoxic phenotype.

### 5.2 Clinical studies across multiple solid tumors demonstrate promising therapeutic potential

#### 5.2.1 Neoadjuvant chemoradiotherapy combined with ICIs and Tα1 for stage II/III colorectal cancer

A single-arm phase II study conducted by Zhang Zhongtao and Yao Hongwei et al. (n=20) evaluated the efficacy of Tislelizumab plus Tα1 in combination with neoadjuvant chemoradiotherapy for locally advanced colorectal cancer (69) (**Table 1**). The Tα1 regimen consisted of 4.8 mg twice weekly (Mondays and Thursdays) for 11 weeks starting from week 1, with Tislelizumab administered during weeks 2, 5, and 8. The objective response rate (ORR) reached 85% (CR 40%, PR 45%), with a disease stabilization rate (SD) of 15%. Only one grade 3 adverse event occurred, while all others were grade 1–2. After a median follow-up of 10 months, the event-free survival (EFS) rate was 100%.

**Table 1:** Summary of clinical evidence.

Case	Research type	N	Cancer	Therapeutic schedule	Prognosis	Adverse reaction
Zhang et al. (69)	Single-arm Phase II	20	Stage II-III colorectal cancer	50Gy/25f Capecitabine 850-mg/m^2^ Tislelizumab 200mg Tɑ1 4.8mg biw	ORR 85%	Grade 2: 75% Grade 3: 5%
Xu et al. (70)	Single-arm Phase II	30	Stage III gastric cancer	Sox Serplulimab 300mg Tɑ1 4.8mg biw	MPR 37.5%	Diarrhea: 46.7% Loss of appetite: 23.3%
Zhang et al. (71)	Single-arm	9	Stage IV melanoma	1 week Tɑ1 1.6mg qd 2–4 week 1.6mg tiw Toripalimab	ORR 22.2% DCR 77.8%	Grade 1: 33.3%
Zhang et al. (72)	Double-arm	a:48 b:101 c:47	Locally advanced non-small cell lung cancer	CCRT Tɑ1 1.6mg qw Nivolumab	mPFS $\left\{ \begin{array}{c} a\;14.6m(11.9-17.3)\\ b\;16.0m(13.2-18.8)\\ c\;not\;reach \end{array}\right.$mOS $\left\{ \begin{array}{c} a\;20.0m(16.1-23.9)\\ b\;27.6m(13.8-41.3)\\ c\;not\;reach\; \end{array}\right.$	Grade 2 pneumonia $\left\{ \begin{array}{c} a\;35.4\%\\ c\;14.5\% \end{array}\;\right.$p=0.01 6m of lymphopenia $\left\{ \begin{array}{c} a\;55.8\%\\ b\;30.9\%\\ c\;22.5\% \end{array}\right.$
Zhang et al. (73)	Single-arm	21	Late-stage recurrent/refractory solid tumor	PD-1/PD-L1 Radiotherapy GM-CSF Tɑ1	ORR 23.8% DCR 47.6%	Not seen at level 3 or above

a: Unused Tɑ1, b: short period of Used Tɑ1 , c: long period of Used Tɑ1; *m:month;* ORR: Objective Response Rate; DCR: Disease Control Rate; MPR: Major Pathological Response; mPFS: median Progression-Free Survival; mOS: median Overall Survival.

#### 5.2.2 Neoadjuvant therapy combining Tα1, Serplulimab, and SOX regimen for HER2-negative stage III gastric cancer

Xu Zekuan and Xu Hao et al. conducted a single-arm trial (n=30) assessing Serplulimab plus Tα1 and SOX chemotherapy as neoadjuvant therapy for gastric cancer (70). The protocol included three cycles of Serplulimab combined with Tα1 (4.8 mg twice weekly). Among 16 patients who underwent surgery after treatment, the pathological complete response (pCR) and major pathological response (MPR) rates were 25% and 37.5%, respectively. Treatment-related adverse events (TRAEs) occurred in 66.7% of patients (mainly diarrhea, decreased appetite, and neutropenia), but the incidence of irAEs was only 10%, with no grade ≥4 events.

#### 5.2.3 Tα1 plus toripalimab in elderly patients with advanced melanoma

Zhang Xiaoshi and Zhou Penghui et al. enrolled nine elderly stage IV melanoma patients with comorbidities and ECOG ≥2 to receive induction and maintenance therapy with Tα1 plus toripalimab (71). The Tα1 regimen consisted of 1.6 mg daily during week 1, followed by 1.6 mg three times per week thereafter. At a median follow-up of 9.9 months, ORR and disease control rate (DCR) were 22.2% and 77.8%, respectively. The median progression-free survival (PFS) was 8.2 months, while median overall survival (OS) had not been reached. All TRAEs were grade 1 (two cases of vitiligo and one of elevated transaminases). Laboratory monitoring revealed decreased neutrophil-to-lymphocyte ratio and increased lymphocyte percentage, indicating immune function restoration.

#### 5.2.4 Salvage therapy combining Tα1 with the BRaG regimen for advanced refractory solid tumors

Zhang Liyuan et al. investigated Tα1 combined with the BRaG regimen (ICI + radiotherapy + G-CSF) in 21 patients with relapsed or refractory solid tumors following second-line therapy failure (73). Tα1 dosing was stratified by baseline lymphocyte count: patients below normal range received loading doses (1.6–3.2 mg daily for 7 days), while others received maintenance (1.6 mg twice weekly). Toripalimab was initiated post-radiochemotherapy and continued until progression. The ORR was 23.8%, DCR 47.6%, and median PFS 3.97 months (95% CI: 2.67–6.97), with no grade ≥3 adverse events. Peripheral immune profiling revealed expansion of CD8^+^ T cells, NK cells, and CD4^+^ effector memory T cells (TEM), alongside a significant reduction in Tregs (P = 0.001), suggesting reversal of T-cell exhaustion by Tα1.

#### 5.2.5 Improved prognosis in unresectable locally advanced NSCLC with concurrent chemoradiotherapy and maintenance nivolumab plus Tα1

Zhang et al. conducted a large-scale retrospective study in locally advanced, unresectable NSCLC patients receiving CCRT followed by nivolumab maintenance, stratified into three subgroups: no Tα1 (n=48), short-term Tα1 (1.6 mg weekly during CCRT; n=101), and long-term Tα1 (continued up to 12 months post-CCRT; n=47) (72). Compared to the non-Tα1 group, both Tα1-treated groups achieved superior median PFS and OS (PFS: 14.6 vs. 16.0 vs. not reached, P = 0.03; OS: 20.0 vs. 27.6 vs. not reached, P = 0.01). The incidence of ≥grade 2 pneumonitis was significantly lower in the long-term group (14.5% vs. 35.4%, P = 0.02). Furthermore, the rate of lymphopenia at 6 months was reduced (22.5% vs. 30.9% vs. 55.8%, P = 0.01), and median IL-6 levels were markedly decreased (4.92 vs. 8.14 pg/mL, P = 0.03), confirming both the safety and long-term immunological benefit of Tα1 co-administration.

### 5.3 Comparison of Tα1 with other immunomodulatory agents

Based on available preclinical and clinical findings, Berry et al. reported that STING agonists combined with ICIs significantly enhanced antitumor efficacy in animal models. In combination with pembrolizumab, notable tumor regression was observed; however, no specific data were provided, and the incidence of adverse events associated with the combination therapy was not addressed. Notably, studies on STING agonist monotherapy have indicated a relatively high frequency of fever, headache, and injection site pain (74). Julie et al. demonstrated that a vaccine targeting IDO and PD-L1 combined with nivolumab increased antitumor efficacy; however, the incidence of adverse events was not reduced compared with nivolumab monotherapy, with grade 3–4 adverse events occurring in 13% of the 30 patients enrolled (75).

Compared with the findings for Tα1, these results further highlight that Tα1 in combination with ICIs not only enhances antitumor efficacy but also ensures a favorable safety and tolerability profile (**Table 2**).

**Table 2:** Comparison of the characteristics of Tα1 with STING agonists and tumor-targeted IDO/PD-L1 therapies.

Name	Target of action	Involved molecular/cellular signaling pathways	Synergistic interaction with ICIs	Effect of irAEs
Tα1	Various immune cell surface receptors, e.g. the TLR family	1. Promotes cytokine release 2. Enhances activation, proliferation, and differentiation of dendritic cells (DCs), macrophages, and natural killer (NK) cells 3. Upregulates expression of MHC class I and class II molecules 4. Directly or indirectly acts on T lymphocytes, promoting their activation, proliferation, and cytotoxic function, while inhibiting apoptosis	Thymosin alpha 1 (Tα1) promotes cytokine release and enhances the function of antigen-presenting cells (APCs) and T lymphocytes. This effect can synergize with immune checkpoint inhibitors (ICIs) to augment NK cell- and T lymphocyte-mediated cytotoxicity against tumor cells, while ICIs concurrently inhibit tumor immune evasion.	Tα1→Pro−inflammatory cytokine release↑ Tα1→Anti−inflammatory cytokine release↓ Tα1→IDO activity↑ Tryptophan catabolism→Treg cell activation and proliferation↑ Tryptophan catabolism→T lymphocyte functional metabolism ↓ The aforementioned immunomodulatory effects contributed to a decreased overall incidence of irAEs
STING agonist	Promotes activation of the STING signaling pathway	cGAMP → STING activation → TBK1 → IRF3 → ISGs(Antiviral genes) ↑ → Type I interferons↑	STING activation triggers a cascade that enhances DC antigen presentation and T lymphocyte homing, promotes infiltration of CD3^+^ and CD8^+^ T cells in the tumor microenvironment (TME), and upregulates MHC class I molecule expression. This effect synergistically coordinates with ICIs to enhance T cell-mediated tumor cytotoxicity.	No mentioned
Targeted IDO^+^/PD-L1^+^ vaccine	Targeting tumor surface IDO^+^/PD-L1^+^ molecules	Specifically targets and eliminates IDO^+^/PD-L1^+^-expressing tumor and immunosuppressive cells	Targeted killing of tumor cells and immunosuppressive cells remodels the immunosuppressive tumor microenvironment and enhances T cell-mediated tumor cytotoxicity promoted by ICIs	Targeted cytotoxicity against IDO^+^/PD-L1^+^ immunosuppressive cells results in a relatively elevated incidence (13%) of severe (grade ≥3) immune-related adverse events.

## 6 Discussion

This review summarizes the molecular mechanisms by which Tα1 modulates the immune system, as well as the findings from existing preclinical and clinical studies. Collectively, the evidence reinforces Tα1’s multifaceted immunoregulatory properties and highlights its synergistic potential when combined with ICIs, yielding enhanced antitumor efficacy and improved treatment safety.

Both preclinical and clinical data collectively offer a promising avenue to address critical bottlenecks in cancer immunotherapy. Mechanistic studies provide compelling evidence that Tα1 enhances ICI safety profiles and potentiates their antitumor efficacy (31). From an immunobiological perspective, Tα1 selectively modulates immune activity across different tissue compartments: it upregulates IDO1 mRNA expression and tryptophan metabolism in non-tumor tissues (31, 47), thereby restraining excessive immune activation, while within TME, it does not elevate IDO1 levels but instead enhances lymphocytic infiltration, boosts effector T-cell function, and reduces Treg accumulation (31, 47). This spatially selective immunomodulation illustrates Tα1’s capacity to augment antitumor immunity without inducing systemic hyperactivation, thereby supporting its immunological safety in combination therapy.

Furthermore, the alignment between clinical endpoints and mechanistic biomarkers corroborates the biological plausibility of Tα1–ICI synergy. Notably, the study by Zhang Xiaoshi and Zhou Penghui et al. (69) reported a DCR approaching 80% and a median PFS exceeding eight months in elderly patients with advanced melanoma, many of whom had significant comorbidities. Importantly, median OS had not yet been reached at 8.3–16.0 months of follow-up, with only mild (grade ≤2) adverse events observed. The associated increase in circulating lymphocytes further supports restoration of immune competence in this vulnerable population. These findings underscore the unique therapeutic value of Tα1–ICI co-administration in geriatric oncology and its broader implications in the context of global population aging.

A previous large-scale phase III randomized controlled trial investigating Tα1 in the treatment of sepsis demonstrated no statistically significant difference in adverse events between the Tα1 and placebo groups. During the 90-day follow-up period, 66% of patients experienced anemia (10.7%), fever (9.6%), abdominal distension (5.4%), and coagulation disorders (4.8%), with no unexpected serious adverse events attributed to Tα1 (76). Findings from a systematic review also indicated that none of the evaluated randomized controlled trials reported serious adverse events or treatment discontinuation associated with Tα1 (77). In prior studies on hepatitis, adverse events related to Tα1 were limited to injection site discomfort and mild fatigue (78). Collectively, these studies provide clear evidence supporting the safety profile of Tα1, which serves as a foundation for the current combination regimen of Tα1 with ICIs. In addition to the improved incidence of adverse events observed with this combination therapy, as discussed in the preceding sections, further validation through large-scale, multicenter prospective randomized controlled trials is warranted.

Integrating evidence from both preclinical and clinical studies, Tα1 has been shown to exert pleiotropic immunomodulatory effects by enhancing both innate and adaptive immunity. In the context of innate immunity, Tα1 upregulates the expression of MHC class I and II molecules, thereby facilitating antigen presentation. It promotes the activation, differentiation, and proliferation of dendritic cells, enhances macrophage chemotaxis and phagocytic activity, and augments the activation, proliferation, and cytotoxic function of NK cells. In addition, Tα1 stimulates the secretion of multiple cytokines, further amplifying innate immune responses. Clinical studies have consistently demonstrated that Tα1 can increase both the number and functional capacity of peripheral blood lymphocytes in patients with malignancies (79, 80). At the level of adaptive cellular immunity, Tα1 directly targets T lymphocytes by activating intracellular signaling pathways, thereby promoting T-cell activation, proliferation, and differentiation. Mechanistically, Tα1 enhances the expression of cytotoxic effector molecules, including perforin and granzyme, in CD8^+^ T cells, leading to improved antitumor activity. Concurrently, it downregulates senescence-associated markers on T cells, inhibits apoptosis, and prolongs T-cell survival. Clinical evidence further indicates that Tα1 administration in cancer patients is associated with increased proportions of CD3^+^, CD4^+^, and CD8^+^ T cells, as well as B cells and naïve T-cell subsets, accompanied by a reduction in the Treg/T-cell ratio, suggesting a shift toward a more effective antitumor immune profile (20, 72, 81).

Although preclinical studies to date have been methodologically rigorous, they remain limited in fully delineating the mechanisms underlying the synergistic antitumor effects. While Tα1 combined with ICIs has consistently been shown to mitigate irAEs, the precise contribution of Tα1 itself to the observed tumor suppression remains uncertain. Previous studies have demonstrated that Tα1 alone can potentiate immune cell function, inhibit tumor proliferation, and promote apoptosis (82, 83). Thus, while the combination therapy clearly outperforms ICI monotherapy, it remains to be clarified whether this superiority extends beyond the intrinsic antitumor capacity of Tα1 monotherapy.

Current clinical findings are indeed encouraging, yet the stability and reliability of these outcomes warrant cautious interpretation. Most existing trials have employed multimodal regimens—typically integrating radiotherapy and/or chemotherapy alongside Tα1 and ICIs—reflecting contemporary oncologic treatment paradigms aimed at enhancing efficacy and minimizing toxicity. However, the inclusion of additional cytotoxic or radiosensitizing agents introduces potential pharmacodynamic interactions that may confound efficacy assessment and inflate response rates via synergistic or additive effects. Consequently, the strength of causal inference for the Tα1–ICI combination alone remains limited, underscoring the need for rigorously designed, high-level evidence studies.

Moreover, the available clinical data are predominantly derived from single-center, small-sample trials, thereby restricting external validity and generalizability. Future investigations should prioritize large-scale, multicenter, randomized controlled studies with well-defined comparator arms (Tα1 + ICI vs. ICI alone vs. Tα1 alone) across diverse tumor types and subtypes. Stratified analyses by age, tumor histology, and molecular profile are also necessary. In addition, due to the influence of pharmacokinetic factors, the therapeutic efficacy of Tα1 in combination with ICIs may vary significantly when administered before, during, or after ICI treatment—an issue that warrants further investigation. The efficacy of any drug is largely dose-dependent. When Tα1 is combined with ICIs for tumor therapy, high-dose administration may potentially induce excessive immune activation in non-tumor tissues, leading to unintended tissue injury, while within TME, it may trigger an excessively potent antitumor response and increase the risk of tumor lysis syndrome. Therefore, it is imperative to design dose-escalation and frequency-optimization studies within the established monotherapy safety range of Tα1 to determine the optimal dosing strategy (e.g., dose level—1.6 mg or higher; administration frequency—twice weekly, three times weekly, or daily; and treatment duration) and to ensure the overall safety and efficacy of the combination regimen. Furthermore, monitoring of efficacy biomarkers, such as changes in lymphocyte subsets, should be implemented. Although the anticipated clinical outcomes of this study are promising, the current high cost of Tα1 presents a challenge. At present, there is a lack of large-sample clinical studies comparing the cost-effectiveness of Tα1 adjuvant therapy versus the treatment costs associated with irAEs resulting from ICI therapy, as well as the subsequent impact on therapeutic outcomes. Given that some patients may have concerns regarding the financial burden, multifaceted efforts in clinical research are warranted to address this issue.

Tα1, as an immunomodulatory agent, has been widely applied in conditions such as sepsis, viral hepatitis, and other infections associated with immune suppression. Through modulation of multiple intracellular signaling pathways, Tα1 enhances innate immune responses while attenuating excessive inflammatory injury, including cytokine storm–related damage. The underlying mechanisms of signal activation and transduction have been progressively elucidated, providing a solid theoretical basis for its clinical use in immune enhancement and regulation. Emerging clinical evidence in oncology has demonstrated that Tα1 administration is associated with significant increases in peripheral blood lymphocyte subsets, further supporting its immunoregulatory effects on T lymphocytes. In conjunction with preclinical findings, Tα1 has been shown to modulate innate immunity, adaptive immunity, and immune tolerance in cancer patients. However, the precise mechanisms by which Tα1 acts within the tumor microenvironment remain incompletely understood. In particular, there is a lack of robust evidence regarding its direct effects on T-cell subsets in solid tumor microenvironments, which limits the overall strength of current evidence. Therefore, to better inform the clinical application of Tα1 in solid tumors, further in-depth mechanistic studies are warranted, especially those focusing on functionally distinct T-cell subsets involved in antitumor immunity.

## 7 Conclusion

Tα1, as a multi-target immunomodulator, can synergistically enhance the anti-tumor effects of ICIs. It not only improves the therapeutic efficacy of ICIs but also reduces the incidence of irAEs and mitigates the toxicity of ICIs, without compromising their anti-tumor activity. Furthermore, Tα1 exerts selective immunomodulatory effects in tissues outside TME. Based on these findings, we conclude that Tα1, when used as an adjunct to ICIs, holds the potential to overcome the current clinical limitations of ICIs in cancer treatment. It could broaden the therapeutic scope of ICIs, improve their safety profile, and bring about another breakthrough in cancer treatment. Moreover, existing clinical studies suggest that the combination therapy holds promise for achieving significant therapeutic benefits even in elderly cancer patients and those with advanced-stage disease. Therefore, large-scale, multi-center clinical trials are urgently needed to further strengthen the evidence supporting combination therapies, providing better guidance for subsequent clinical practice.
